# The Three Breath Method: Teaching Coping Mechanisms for Processing Patient Death

**DOI:** 10.5070/M5.53087

**Published:** 2026-07-31

**Authors:** Sarah Petelinsek, Genae Christensen, Alexander Franke, Christine Raps, Allison Beaulieu, Megan Fix, Gabrielle Langmann, Patrick G Hughes

**Affiliations:** * Spencer Fox Eccles School of Medicine at the University of Utah Health, Salt Lake City, UT; ^ University of Utah Health, Department of Emergency Medicine, Salt Lake City, UT; † University of Utah Health, Department of Internal Medicine, Salt Lake City, UT

## Abstract

**Audience:**

The target audience for this video is any provider who is likely to experience patient death. We have demonstrated this method to medical students, residents, and faculty and across departments and disciplines of health care including physician assistants and nurses.

**Introduction:**

Emergency medicine (EM) physicians frequently encounter patient death, requiring effective coping strategies. Brief mindfulness practices, including focused breathing, can promote emotional regulation and reduce perceived stress. These techniques are particularly relevant in acute care settings where rapid cognitive and emotional transitions are required.^1^ While techniques such as “The Pause”—a moment of silence after death—have been proposed, their impact remains understudied. 2–4 In addition to the Pause Method, many physicians at our institution utilize the “Three-Breath Guided Meditation” technique, a structured deep-breathing exercise designed to help reduce stress. This study aimed to assess EM physicians’ perceived ability to process patient death, explore coping strategies, and evaluate the effectiveness of both “The Pause” and the “Three-Breath Method.”

**Educational Objectives:**

After watching the video, proposed viewers should be able to: 1) describe the Three-Breath technique, and its potential impact on healthcare providers’ ability to manage stress and process patient death, 2) evaluate the effectiveness of the Three-Breath guided meditation in improving emotional reconciliation and reducing stress for emergency medicine physicians, and 3) demonstrate the Three-Breath technique and implement it into their own practice.

**Educational Methods:**

Emergency medicine residents and faculty were invited via email to watch a 4-minute instructional video on the Three-Breath Technique. This video lecture was made available asynchronously, and participants were encouraged to watch the video at a time that was suitable to them.

**Research Methods:**

Emergency medicine residents and attending physicians completed a mixed-methods questionnaire on their coping abilities after patient death. The survey included baseline experiences, coping practices, comfort levels, and the 4-minute instructional video. Responses were analyzed using descriptive statistics, Spearman’s correlation for continuous variables, and thematic analysis of free-text data.

**Results:**

Of 79 invited EM physicians, 49 responded (62%), including 39% attendings and 61% residents (median age 33.5 years, 53% male, 47% female). A weak correlation was found between training level and perceived ability to process death (r=0.27), with attending physicians reporting greater comfort (mean = 4.45) than residents (mean = 3.75). Perceived ability to process death moderately correlated with comfort returning to work (r=0.55). Overall, 59% used the Pause or Three-Breath techniques, and 94% reported comfort coping after patient death.

**Discussion:**

Physicians’ comfort in processing patient death increases with training level, with attendings feeling more prepared than residents. Most providers use the Pause^1^–^3^ or Three-Breath Method and report comfort in coping. However, the study’s single-institution scope, modest sample size, and reliance on self-reported data may limit generalizability. Future efforts should be expanded to additional specialties and emphasize flexibility and individualized strategies to support emotional well-being in clinical practice.

The Three-Breath guided meditation technique, as an extension of The Pause,^2^–^4^ is being widely adopted by physicians at one US academic institution and may enhance physicians’ ability to process patient death, promoting emotional reconciliation and stress reduction in high-pressure healthcare environments.

**Topics:**

Physician coping with patient death, coping strategies, novel coping strategy, emotional resilience.

## USER GUIDE

**Table:** 

List of Resources:	
Abstract	1
User Guide	3
Appendix A: Lecture Video Link	5
Appendix B: Group Discussion Facilitator Guidelines	6

**Learner Audience:**

Medical students, Residents, Faculty, Physician Assistants and Nurses

**Time Required for Implementation:** Four minutes to complete the entirety of the video. If watching in a group session, we recommend a 1–minute introduction to the content, and a 5–10 minute group discussion following the session. This would total 10–15 minutes.

**Recommended Number of Learners per Instructor:**

There is no minimum or maximum. This training is appropriate for large group, small group, or individual use.

**Topics:**

Physician coping with patient death, coping strategies, novel coping strategy, emotional resilience.

**Objectives:**

After watching the video, proposed viewers should be able to:

1. Describe the Three-Breath technique, and its potential impact on healthcare providers’ ability to manage stress and process patient death.
2. Evaluate the effectiveness of the Three-Breath guided meditation in improving emotional reconciliation and reducing stress for emergency medicine physicians.
3. Demonstrate the Three-Breath technique and implement it into their own practice

### Linked objectives and methods

Based on The Pause, 2–4 we utilized a 4-minute asynchronous video lecture to teach a novel strategy of coping with patient death. Following Mayer’s cognitive theory of multimedia learning,^5^ the video-based instructional content was developed to minimize extraneous load and promote meaningful learning. This method was chosen because of its wide dissemination capabilities. By utilizing a video lecture format, we were able to offer this teaching on a much wider scale with very few logistical hurdles. This video format allowed us to distribute this content to medical students, medicine residents, and medicine faculty, as well as to physicians assistants and nurses.

Additionally, it allowed for cross-departmental dissemination of the work in internal medicine and critical care across multiple hospital centers.

### Link to Lecture

- https://youtu.be/qzLKGILD6uc

### Results and tips for successful implementation

The initial teaching of the Three Breath Method occurred organically at our institution. A chief resident began implementing this in his practice, and many of the rest of the residency and faculty then modeled this young physician’s behavior. We recorded this teaching to allow this Three Breath Method to continue to be taught beyond the emergency medicine department and outside of the institution beyond the graduation of the chief resident. The intention of this video lecture was to allow participants the opportunity to think about their current coping mechanism and offer the Three Breath Method as a potential first step or additional tool in their coping journey.

Of 79 physicians invited to participate in the survey, 49 responded (62%), including 39% attendings and 61% residents (median age 33.5 years, 53% male, 47% female). A weak correlation was found between training level and perceived ability to process death (r=0.27), with attending physicians reporting greater comfort (mean = 4.45) than residents (mean = 3.75). Perceived ability to process death moderately correlated with comfort returning to work (r=0.55). Overall, 59% used the Pause or Three-Breath techniques, and 94% reported comfort coping after patient death.

### Technology necessary

This video lecture requires internet access to view the video content. Ideally the user would have audio as well as visual capabilities.

## Supplementary Information



## Figures and Tables

**Figure:**
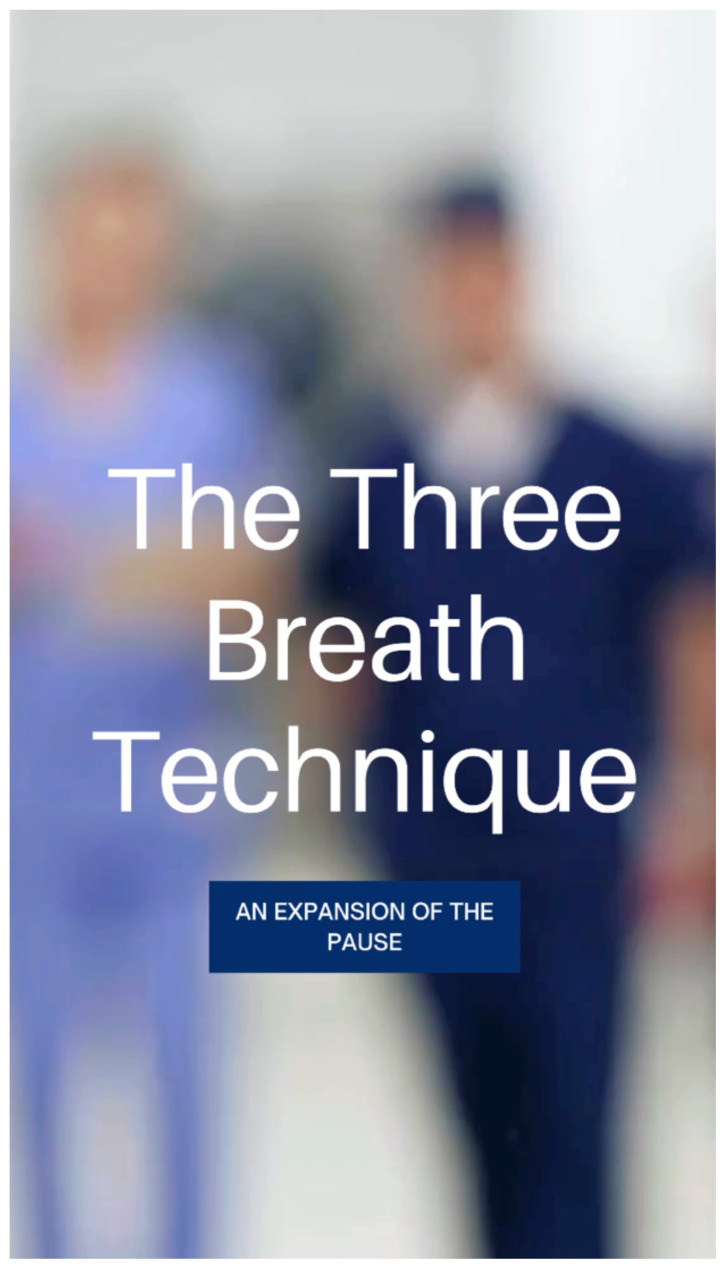
Please see associated video file Video Link: https://youtu.be/qzLKGILD6uc

## Appendix B Group Discussion Facilitator Guide

Talking point suggestions:

- Do you have a personal ‘reset’ after a bad case?
  ○ What about something in that moment vs after your shift?
- Is coping something we should formalize? Or keep personal? Why?
  ○ This didn’t start as a protocol; it started as a human response.
  ○ Sometimes people don’t know where to start, though, and giving protocol may be better than nothing
- Why does this technique work? (It’s practicable, portable, etc.)
- Would something this simple actually change behavior in your department?
